# Comparison of positivity frequency of bcl-2 expression in prostate adenocarcinoma with low and high Gleason score

**DOI:** 10.1590/S1516-31802001000400005

**Published:** 2001-07-07

**Authors:** Flávio Luiz Ortiz Hering, Mônica Vannucci Nunes Lipay, Marco Aurélio Silva Lipay, Paulo Roberto Teixeira Rodrigues, Luciano José Nesralah, Miguel Srougi

**Keywords:** bcl-2, Prostate, Prostate adenocarcinoma, Apoptosis, bcl-2, Próstata, Adenocarcinoma da próstata, Apoptose

## Abstract

**CONTEXT:**

Multiple genetic and epigenetic factors have been implicated in the oncogenesis and progression of prostate cancer. The major difficulty is in that the clinical management stems from the reality that reliable and accurate prognostic biomarkers are not available and that effective treatment regimens forming hormone-resistant prostate cancers are yet to be developed. Among the most important regulators of apoptosis and programmed cell death is the bcl-2 gene and its related proteins. Elevated levels of bcl-2 protein may contribute to the progression of prostate cancers to a metastatic and hormone-insensitive state characterized by poor responses to chemotherapy.

**OBJECTIVE:**

To characterize the expression of bcl-2 proteins as a prognostic factor in humans.

**DESIGN:**

A retrospective approach.

**SETTING:**

Urologysection, Federal University of São Paulo.

**DIAGNOSTIC TEST USED:**

Immunohistochemical analysis using bcl-2 protein antibody and normal staining by hematoxylin-eosin.

**MAIN MEASUREMENTS:**

Prognostic relations and protein expression were evaluated considering the total sample (28) divided into two groups, high (8 to 10) and low (2 to 4), separated according to the histological differentiation grade (Gleason score) with 10 and 18 samples, respectively.

**RESULTS:**

The differentiation of grade into two groups separated according to the Gleason score in low and high types presented different bcl-2 expression (P < 0.001).

**CONCLUSION:**

The higher frequency of bcl-2 immunostaining in tumor samples was observed in association with more advanced Gleason scores and suggests that an increase in the ratio of this anti-apoptotic protein often occurs during progression of prostate cancers.

## RESUMO

**CONTEXTO:**

Diversos fatores genéticos e ambientais têm sido relacionados à oncogênese e progressão do câncer de próstata. A principal dificuldade na conduta clínica frente a esses tumores consiste no fato de não existirem marcadores prognósticos precisos, e que tratamentos eficazes dos tumores hormônio-resistentes não estão ainda disponíveis. Entre os reguladores mais importantes para avaliar a taxa de apoptose, destaca-se a expressão da proteína bcl-2. Os níveis elevados dessa proteína podem contribuir na progressão dos tumores prostáticos para um estado metastático e/ou hormônio independente, que caracteristicamente não apresenta resposta satisfatória ao tratamento quimioterápico.

**OBJETIVO:**

Investigar a expressão da proteína bcl-2 como fator prognóstico em portadores de tumores prostáticos.

**TIPO DE ESTUDO:**

Estudo retrospectivo.

**LOCAL:**

Disciplina de Urologia da Universidade Federal de São Paulo.

**TESTE DIAGNÓSTICO:**

Exame anatomopatológico convencional por hematoxilina-eosina (HE) e imunohistoquímico.

**VARIÁVEIS ESTUDADAS:**

Expressão prognóstica da proteína bcl-2, por técnica imunohistoquímica em portadores de adenocarcinoma da próstata submetidos a prostatectomia radical.

**AMOSTRA:**

28 amostras de tumores de próstata fixadas em parafina, provenientes de pacientes submetidos à prostatectomia radical sem ablação androgênica prévia, avaliadas por HE e por imunohistoquímica para a caracterização da expressão da proteína bcl-2. A expressão foi correlacionada ao grau de diferenciação do tumor, dividindo-se os tumores em dois grupos, segundo o escore de Gleason, em baixo (escore 2 a 4), e alto grau (8 a 10), sendo que os grupos foram divididos em 18 e 10 pacientes, respectivamente.

**RESULTADOS:**

Observou-se associação estatisticamente significativa (P < 0, 001) entre expressão elevada da proteína e os casos que apresentavam um escore de Gleason mais elevado (83, 3%), enquanto que no grupo de escore de baixo grau não foi detectada expressão da bcl-2 em 90% das amostras.

**CONCLUSÕES:**

O aumento na expressão da proteína bcl-2 em tumores de próstata com escore de Gleason elevado pode estar associado à progressão do adenocarcinoma de próstata.

## INTRODUCTION

Adenocarcinoma of the prostate is one of the most common malignancies in men and one of the main causes of cancer death.^1^^,^^2^ In spite of its high incidence, prostate cancer has a very heterogeneous biological behavior, and therefore it is very difficult to define its evolution.^3^ The prognosis of these tumors depends on some parameters such as the TNM staging, the histological differentiation, and others. Today, the discovery of genetic abnormalities has an important role in defining the prognosis of cancer.^2^

Among the most important regulators of apoptosis and programmed cell death are bcl-2 and its related proteins. The bcl-2 gene was first discovered because of its involvement in the t(14, 18) chromosomal translocations commonly found in non-Hodgkin's follicular B cell lymphomas.^1^ The protein encoded by this gene is a potent blocker of apoptosis and resides primarily in the outer mitochondrial membrane, nuclear envelope, and parts of the endoplasmic reticulum.^4-6^

The bcl-2 gene product regulates programmed cell death^7-9^ and a number of studies suggest that bcl-2 is involved in the selection and maintenance of long-living cells, rescuing them from apoptotic cell death, and leading to their accumulation in the G1 phase of the cell cycle.^5^ Abnormalities in the expression of apoptosis suppressing gene bcl-2 are frequently detected in many epithelial neoplasms.^8,10^ The prognostic significance of the abnormal expression of the bcI-2 protein has been analyzed, particularly in breast cancer, and the results suggest that bcl-2 protein expression may also have prognostic significance.^7^^,^^11^

Overproduction of the bcl-2 protein occurs in a wide variety of human cancers and presumably contributes to neoplastic expansion by prolonging cell survival through suppression of the physiological cell turnover mechanisms that normally maintain a homeostatic balance between cell production during cell division and cell loss through programmed cell death.^2^^,^^3^^,^^10^

The bcl-2 is a 25KD oncoprotein expressed in hormone-sensitive epithelium, including the glandular prostatic epithelium. Recent studies have shown the importance of studying this protein in prostate cancer to try to prevent disease development. bcl-2 is expressed in several epithelial tissues,^2^^,^^3^^,^^9^^,^^12^^,^^13^ including the prostatic epithelium.^2^^,^^3^ It is expressed in normal prostatic epithelium, in prostatic ducts and in prostatic tumors, as reported previously.^13^ The expression is more common in tumors that exhibit malignant features, such as the invasive growth type and high proliferation rate of cancer cells, although a previous analysis of a small cohort of cases has suggested no significant relationship between histological differentiation and the expression of bcl-2 protein.^13^

Immunohistochemical analysis of bcl-2 protein levels in prostate tumors has revealed a correlation between the presence of bcI-2 protein and resistance to anti-androgen therapy.^2^ Taken together, these observations suggest that elevated levels of bcl-2 protein may contribute to the progression of prostate cancers to a metastatic and hormone-insensitive state characterized by poor responses to chemotherapy. Using an immunohistochemical approach, Krajewska et al. (1996)^1^ characterized the expression of the bcl-2 family of genes in human prostate cancers and correlated these results with the Gleason grade. The higher levels of bcl-2 immunostaining generally seen here in association with more advanced Gleason grade and metastatic disease suggest that an increase in the ratio of these anti-apoptotic proteins often occurs during progression of prostate cancers.^1^ The intensity of bcl-2 immunostaining and the percentage of immunopositive cells are generally higher in the more advanced tumors, largely accounting for the higher composite scores.^1^

The present analysis was done to test the possible prognostic value of abnormal expression of the bcl-2 protein in prostatic adenocarcinoma associated with the Gleason score, which was divided into two extreme groups: a low score (2-4) and a high score (8-10). This was done with no use of androgen ablation.

## METHODS

We randomly selected 28 patients with a diagnosis of clinically localized adenocarcinoma of the prostate, who underwent radical prostatectomy and were divided into two groups, according to the Gleason score: a) 18 patients with Gleason score 8 to 10, and b) 10 patients with Gleason score 2 to 4. These patients had not been submitted to any kind of previous hormonal treatment.

### Histological and immunohistochemistry methods.

First, 5 µm-thick sections were cut from the paraffin-embedded tumor specimens and stained with HE. The samples were examined for the histological prognostic factors by one investigator, who was unaware of the clinical data. Tumors were graded according to Gleason's grading system. All slides were reviewed and a Gleason score was determined by adding the numbers for the two most predominant patterns.

For the immunohistochemical demonstration of the bcl-2 protein, 5-µm sections from the primary prostatic carcinomas were deparaffinized, rehydrated and washed for 5 minutes with PBS. Thereafter the sections were rinsed in distilled water and heated in a microwave oven for 15 to 45 minutes in 0.01 M citrate buffer (pH 6.0). After microwave oven heating, the slides were rinsed in Tris-buffered saline (pH 7.4). Endogenous peroxide was blocked by 3% hydrogen peroxide for 5 minutes, followed by washing for five minutes with PBS. The tissue sections were incubated with the monoclonal anti-bcl-2 protein (Dako Corp., CA) antibody diluted at 1:400 in PBS. Sections were washed twice for 5 minutes with PBS, and incubated for 20 minutes with AB Duet complex (Dako Corp., CA) diluted at 1:200 in PBS for 30 minutes. Sections were washed twice for five minutes with PBS, developed with diaminobenzidine tetrahydrochloride substrate (Sigma), slightly counterstained with hematoxylin, dehydrated, cleared and mounted.

### Scoring of bcl-2 expression.

All slides were evaluated for immunostaining in a blind fashion, without any knowledge of the clinical outcome or other clinical and pathological data. The proportional pattern and intensity of immunostaining for bcl-2 were evaluated.

### Statistical methods.

The comparison of proportions of immunostaining of the oncoprotein bcl-2 between the two different Gleason score groups was done using the Mann-Whitney test.

## RESULTS

A total of 28 cases of prostate adenocarcinoma, consisting of 10 cases classified as low Gleason score (2, 3, 4) and 18 cases of high Gleason score (8, 9, 10), were examined in this study. The Gleason score and frequency of cells immunostained for bcl-2 in each group are summarized in Tables 1 and 2.

**Table 1:** Gleason score and bcl-2 staining frequency in patients with adenocarcinoma of the prostate

High Gleason Score		
Patient	Gleason	bcl-2 staining (%)
1	8	70
2	8	80
3	9	65
4	8	35
5	9	70
6	8	75
7	9	85
8	10	95
9	10	90
10	9	85
11	8	90
12	8	70
13	8	35
14	8	75
15	8	85
16	8	90
17	9	95
18	9	75

**Table 2:** Gleason score and bcl-2 staining frequency in patients with adenocarcinoma of the prostate

Low Gleason Score		
Patient	Gleason	bcl-2 staining (%)
1	4	10
2	4	20
3	4	30
4	4	25
5	3	15
6	4	10
7	4	10
8	4	15
9	4	15
10	4	80

**Figure 1:**
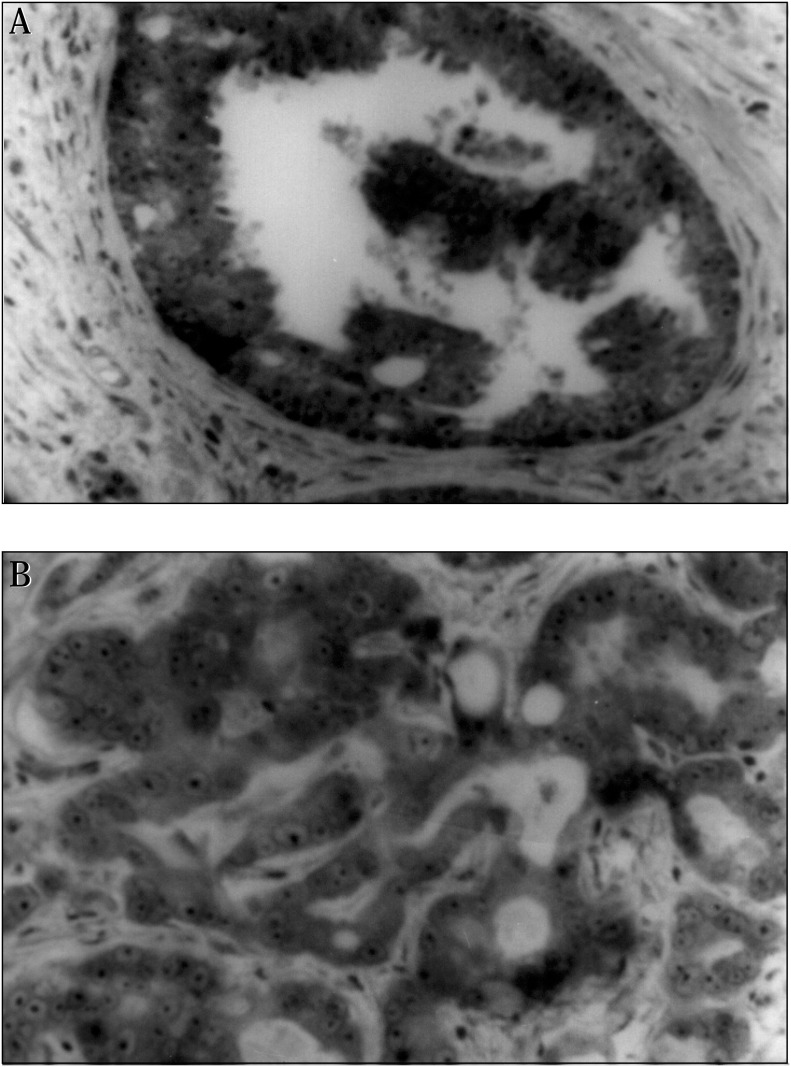
Immunohistochemical analysis of bcl-2 protein in prostate cancers. Representative photomicrographs are shown of tissue sections derived from primary prostate cancers that were immunostained using antibodies specific for bcl-2. Antibody detection was accomplished using a diaminobenzidine colorimetric method (brown) and nuclei were counterstained with hematoxylin (blue). a) High grade (Gleason 8-10) tumor with a bcl-2 positive high immunoreactivity (40X).b) Low grade (Gleason 2-4) tumor illustrating an example of a mostly bcl-2 negative expression (100X).

Of the 18 cases of high Gleason score (Table 1) 15 had a staining frequency of 70% or more (Figure 1a). Considering the low Gleason group (Table 2), 9 cases of a sample of 10 presented a staining of less than 30% of the cells expressing the bcl-2 protein (Figure 1b). One patient had an 80% staining frequency in this group, probably due to other factors of poor prognosis simultaneous to bcl-2 expression and not identified in this approach. This difference between the two groups was statistically significant (P < 0.001) indicating an over-expression in patients of the group presenting a higher Gleason score.

## DISCUSSION

Prostate cancer, like most other solid tumors, represents a very heterogeneous entity. Most prostate cancers, at the time of clinical diagnosis, present themselves as mixtures of androgen-dependent and androgenindependent cells.^2^ Most prostate cancers respond initially to androgen ablation since the population of androgen-dependent cells undergoes rapid apoptosis upon androgen withdrawal. However, androgen ablation rarely cures patients, most of whom will experience recurrence due to takeover of the tumor mass by androgen-independent tumor cells as well as the emergence of apoptosis-resistant clones as a result of further genetic alterations such as bcl-2 amplification.^14^

Proteins encoded by the bcl-2 family of genes are important regulators of programmed cell death and apoptosis.^3^ Alterations in the expression of these apoptosisregulating genes can contribute to the origins of cancer, as well as adverse tumor responses to chemotherapy and radiotherapy.^1^

Apoptosis has a fundamental role in promoting the tumorigenesis process.^3^ Usually apoptosis is an active non-inflammatory process of cell death that maintains homeostasis. When some abnormalities occur, the process is facilitated.^1^

bcI-2 is a potent apoptosis-suppressing protein.^15^ This protein has a well-known role in developing some lymphomas and perhaps in the progression of breast and prostate cancer, by maintaining the longevity of cells via suppressing their programmed death.^2^^,^^16^ Its expression is more common in tumors with invasive behavior, high proliferation rate and abnormal cell differentiation features.^2^

We observed a significant difference in the bcl-2 protein expression between the two groups of patients with adenocarcinoma of the prostate. This variation could be due to different characteristics of the disease evolution. Westin et al. (1995),^17^ studying biopsies of prostate cancer observed that bcl-2 increased one week after castration and after androgen ablation before tumor regrowth. Tsuji et al. (1998)^1^ verified that bcl-2 expression increases with androgen ablation and the proliferative activity of tumor cells is significantly reduced. The bcl-2 expression probably exerts a role in the longevity of tumor cells submitted to hormonal therapy. They also observed that mib-1 expression is related to the different grades of tumor cells, but this does not occur with bcl-2. This data in the literature only considers the expression of these genes after endocrine therapy and this could be an important factor for changing the results.

Colombel et al. (1993),^13^ studying bcl-2 expression in hormone-resistant prostatic adenocarcinoma, did not correlate the expression of this oncogene with tumor histological differentiation. This difference must probably be due to previous hormone-deprivation treatment, which could alter the interpretation of the histological grade and also the expression of the gene.

There is evidence that some members of the bcl-2/cd9 family regulate programmed cell death in an evolving manner concerning prostate cancer and an over-expression of bcl-2 is associated with progression and an androgenresistant phenotype.^19^

Krajewska et al. (1996),^1^ studying the expression of the bcl-2 family in prostate cancer (genes bax, bcl-x and mcl-1) correlated to the Gleason score, verified that the stage progression and the score increase are positively related to the increase in the expression of these genes. The over-expression of bcl-2 can also give a replication capacity to the hormone- resistant prostatic tumor cells,^20^ associated with the poor prognosis of this neoplasm.^2^

Grossfeld et al. (1998)^19^ observed that, in patients with increased Ki-67, p53 and bcl-2 expression, the recurrence rate for prostate cancer after surgery or radiotherapy was higher.

## CONCLUSION

In conclusion, the over-expression of bcl-2 is significantly higher in patients with an initially elevated Gleason score (8, 9 and 10). Further studies, including intermediate Gleason scores (5, 6 and 7) could provide more evidence for the role of this protein in the development and progression of prostate cancer.
